# Efficacy of Statins in Postmenopausal Osteoporosis: A Systematic Review and Meta-Analysis

**DOI:** 10.7759/cureus.86425

**Published:** 2025-06-20

**Authors:** Pritam Biswas, Sukumar Thorenoor Kumaraswamy, Rob Hilgers

**Affiliations:** 1 Medical Pharmacology, St. Matthews University School of Medicine, Georgeotown, CYM; 2 Medical Microbiology, St. Matthews University School of Medicine, Georgetown, CYM; 3 Medical Pharmacology, St. Matthews University School of Medicine, Georgetown, CYM

**Keywords:** bone mineral density, fracture risk, meta-analysis, postmenopausal osteoporosis, statins

## Abstract

Osteoporosis is a prevalent metabolic bone disease in postmenopausal women, characterized by reduced bone mineral density (BMD) and increased fracture risk. Statins, commonly prescribed for cardiovascular conditions, have been suggested to possess bone-modulating effects via the mevalonate pathway. This systematic review and meta-analysis evaluated the efficacy of statins in improving BMD and reducing fracture risk in postmenopausal osteoporosis. A comprehensive literature search identified six randomized controlled trials involving 1,032 participants. Primary outcomes included changes in BMD at the lumbar spine, femoral neck, and forearm, along with bone turnover markers and fracture incidence. Pooled analyses showed no significant improvement in lumbar spine BMD (mean difference (MD): 13.72%; 95% CI: -19.31 to 46.74; p = 0.42; I² = 100%) or femoral neck BMD (MD: -0.18%; 95% CI: -0.46 to 0.10; p = 0.20; I² = 99%). A statistically significant 1.1% increase in forearm BMD was observed at 52 weeks (p = 0.01), though this effect was based on a single study and lacked generalizability. Bone turnover marker findings were inconsistent, and none of the studies demonstrated significant reductions in fracture risk. The quality of evidence was rated moderate to low due to high heterogeneity and methodological limitations. Overall, statins appear to have limited and site-specific effects on BMD, with insufficient evidence to support their use as a primary therapy for osteoporosis. However, their adjunctive role may be considered in select patients with concomitant cardiovascular disease. Further research is needed to determine long-term benefits, fracture prevention potential, and cost-effectiveness in this population.

## Introduction and background

Osteoporosis is the most common metabolic bone disease. An imbalance in bone turnover lies at the root of its pathogenesis, i.e., a relatively higher bone resorption than bone formation rate, leading to bone mass loss and low-energy fractures [1]. Postmenopausal osteoporosis, resulting from estrogen deficiency, is the most common type of osteoporosis. Estrogen deficiency results in an increase in bone turnover owing to effects on all types of bone cells. The imbalance in bone formation and resorption has effects on trabecular bone (loss of connectivity) and cortical bone (cortical thinning and porosity) [2]. With increasing life expectancies, the number of women at risk of postmenopausal fractures and decreased quality of life would increase as well [3].

The key treatments for osteoporosis are antiresorptive drugs such as bisphosphonates, hormone replacement therapy, raloxifene, calcitonin, denosumab, as well as anabolic hormone analogs of parathyroid hormone (PTH), such as teriparatide. Statins are mainly used for the prevention and treatment of atherosclerosis and cardiovascular disease; however, as these drugs do have an effect on mechanisms associated with bone formation. The use of statins is known to reduce osteoporosis and fractures [4,5].

The rationale behind the use of statins to reduce osteoporosis and fractures stems from reports showing that statins can stimulate the production of bone morphogenetic protein-2 (BMP-2), which promotes the differentiation of osteoblasts, the cells responsible for bone formation [6]. Secondly, statins, being hydroxymethylglutaryl (HMG)-coenzyme A (CoA) inhibitors, interfere with the mevalonate pathway by inhibiting the formation of farnesyl pyrophosphate, similar to anti-resorptive drugs like bisphosphonates, and cause osteoclast apoptosis by inhibiting an enzyme. Intermediary products of this pathway are necessary for the linkage of lipid moieties to cytosolic proteins (protein prenylation), and these linkages are necessary for normal osteoclast function. Thirdly, statins have been shown to inhibit inflammatory cytokines, such as tumor necrosis factor-alpha (TNF-α), IL-6, and C-reactive protein, which can activate osteoclasts [7]. These observations suggest that statins may promote osteoblast activity and inhibit osteoclast activity [8].

A few observational studies have reported a reduced risk of fractures and an increase in bone density in patients on statin treatments [9,10], whereas other studies were inconclusive [11,12] or have found no association [5,13].

Some studies mention that this effect is dose-dependent, and the type of statin, with high-dose lipid-soluble statins having a higher risk of fractures, and water-soluble statins having a lower risk of fractures [14]. In contrast, another study on the same issue claimed that high-dose statins have a protective effect on hip fracture [12]. There is a need to explore the use of the most effective statin at the most effective dose for the treatment of osteoporosis and reduction of the risk of fractures. Many meta-analyses have studied the efficacy of statins on osteoporosis with various conclusions and perspectives [5,10,15-17]. However, the key limitation of the previous meta-analyses was that the evidence towards the efficacy of statins to treat post-menopausal women was inconclusive.

In this report, we will provide a network meta-analytic review of all accessible published investigations purporting the use of adjuvant statins for the treatment of osteoporosis in post-menopausal women. The aim of our current network meta-analysis is to evaluate the efficacy of statins added to standard therapies in the treatment of osteoporosis in post-menopausal women.

## Review

Methods

The protocol for this systematic review and meta-analysis was registered with the International Prospective Register of Systematic Reviews (PROSPERO), NHS National Institute of Health and Care Research, United Kingdom, record number CRD42022339874.

The primary databases that were searched included MEDLINE (via PubMed), Cochrane Central Register of Clinical Trials (CENTRAL), EBSCO Host, Database of Abstracts of Reviews of Effects (DARE), and Google Scholar. Mesh terms and keywords representing participant (P; “osteoporosis, postmenopausal”, “postmenopausal bone loss”, “osteoporosis, "postmenopausal”, Intervention (I; “Statins”, “HMG CoA reductase inhibitors” and Outcomes (O; “fracture”, “BMD”, “adverse event” and “bone turnover markers”). Only Randomized Clinical Trials were included in the study. All synonyms of the keywords were searched using the Boolean operator OR, while the ‘AND’ operator was used to combine terms from different categories. The sample search strategies adapted for databases were detailed in Table 1. This search was supplemented by hand-searching of relevant references from review articles and other eligible studies. No limits were applied to the year of study, and only studies/abstracts available in English were included in the present review.

**Table 1:** PubMed Search Strategy

Search Number	Query	Search Details	Results
3	(("osteoporosis"[All Fields]) OR ("osteoporosis"[MeSH Terms])) OR ("osteoporosis, postmenopausal"[MeSH Terms])	"osteoporosis"[All Fields] OR "osteoporosis"[MeSH Terms] OR "osteoporosis, postmenopausal"[MeSH Terms]	102031
5	(("fracture"[All Fields]) OR ("bone loss"[All Fields])) OR ("bmd"[All Fields]) OR ("bone mineral density"[All Fields])	"fracture"[All Fields] OR "bone loss"[All Fields] OR "bmd"[All Fields] OR "bone mineral density"[All Fields]	311748
6	(("statins"[All Fields]) OR ("hmg coa reductase inhibitors"[All Fields])) OR (statins[MeSH Terms])	"statins"[All Fields] OR "hmg coa reductase inhibitors"[All Fields] OR "hydroxymethylglutaryl coa reductase inhibitors"[MeSH Terms]	49217
7	Search Numbers #3 AND #5 AND #6	("osteoporosis"[All Fields] OR "osteoporosis"[MeSH Terms] OR "osteoporosis, postmenopausal"[MeSH Terms]) AND ("fracture"[All Fields] OR "bone loss"[All Fields] OR "bmd"[All Fields] OR "bone mineral density"[All Fields]) AND ("statins"[All Fields] OR "hmg coa reductase inhibitors"[All Fields] OR "hydroxymethylglutaryl coa reductase inhibitors"[MeSH Terms])	183

In the meta-analysis, RCTs that were available in full text and those that were available as abstracts only were included. Our inclusion criteria included RCTs comparing the efficacy of statins or HMG CoA reductase inhibitors in the treatment of osteoporosis in postmenopausal women. Trials comparing standard treatment protocols with an adjuvant statin (intervention group) with the same regimen without statin (control group) were included. Complete treatment periods of at least 2 months, regardless of the therapeutic dose and method of delivery, were considered. Women of postmenopausal age with a diagnosis of osteoporosis were included. According to WHO, the diagnosis of osteoporosis requires a T score below -2.5. A T score between -1 and -2.5 with a diagnostic fracture risk score called Fracture Risk Assessment tool (FRAX score) of 20% or higher 10-year probability of fracture, or a FRAX score of 3% or higher 10-year probability of hip fracture.

The primary outcomes assessed in this study included the incidence of overall fractures and the percentage change in bone mineral density (BMD) at the lumbar spine, femoral neck, and hip. The RCTs included in the analysis primarily used dual-energy X-ray absorptiometry (DXA) scans to measure BMD. Secondary outcomes focused on changes in anabolic bone markers, such as bone specific alkaline phosphatase (BSALP), a significant marker of bone formation; osteocalcin, a specific marker for bone matrix synthesis; as well as resorptive markers, including C-telopeptide of type I collagen (CTX) (elevated levels CTX suggest increased bone breakdown, which can be associated with conditions like osteoporosis and osteopenia).

All three authors independently screened the identified databases, reviewed abstracts for suitability, and obtained full-text articles for studies deemed eligible or inconclusive based on the abstract screening. A pre-tested data extraction form was created (see Appendices), and all three authors independently extracted the following data from each eligible study: trial site, year, trial methods, participants, intervention, and outcomes. Disagreements between the authors were resolved through discussion.

The extracted data were analyzed using the non-Cochrane mode in RevMan 5.0 software [18]. The heterogeneity between the studies was assessed visually using forest plots; I2 statistics, where more than 50% was considered to have moderate to severe heterogeneity; and the chi-square tests, with a p-value of less than 0.05 indicating statistical significance. Random-effect models were used in cases of moderate heterogeneity.

The methodological quality of six trials was independently assessed by all three authors using the Cochrane Collaboration's tool for assessing the risk of bias. We followed a defined guidance to assess whether trials took adequate steps to reduce the risk of bias across six domains: sequence generation, allocation concealment, blinding (of participants, personnel, and outcome assessors), incomplete outcome data, selective outcome reporting, and other sources of bias. The judgment was categorized into low, high, or unclear risk of bias [19]. The studies included in the meta-analysis were in accordance with the Preferred Reporting Items for Systematic Reviews and Meta-Analyses (PRISMA) guidelines [20]. The grading of the quality of included studies was carried out as per Cochrane's grading of recommendations assessment, development, and evaluation tool (GRADE) [21].

Results

**Figure 1:**
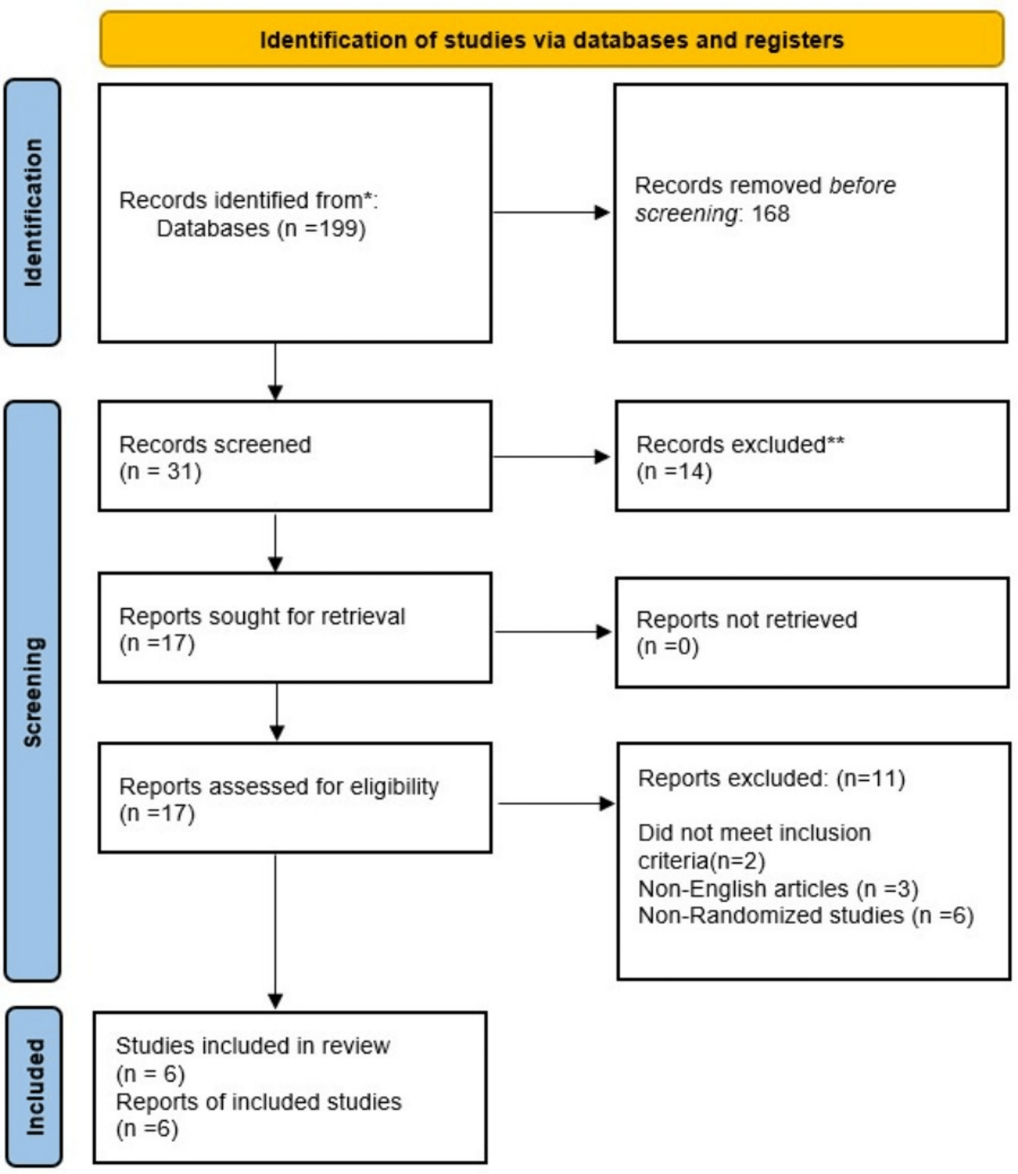
PRISMA flow chart of included studies. PRISMA, Preferred Reporting Items for Systematic Reviews and Meta-Analyses

The systematic search yielded 199 records, of which 168 were excluded during initial screening due to duplication or irrelevance. After assessing 32 records for relevance, 17 reports were sought for eligibility. Following exclusions based on predefined inclusion and exclusion criteria, such as study design, language, and population, six randomized controlled trials meeting the inclusion criteria were included in the final analysis [22-27], as outlined in the PRISMA flow diagram in Figure 1. Table 2 describes the summary of the included studies.

**Table 2:** Summary of Included Studies BMD, bone mineral density; LDL-C, low-density lipoprotein cholesterol; DXA, dual-energy X-ray absorptiometry; QCT, quantitative computed tomography; BSALP, bone-specific alkaline phosphatase; OC, osteocalcin; NTx, N-terminal telopeptide of type I collagen; CTx, C-terminal telopeptide of type I collagen; P1NP, N-terminal propeptide of type I procollagen; DPD, deoxypyridinoline; U-CTX, urinary C-terminal telopeptide; S-CTX, serum C-terminal telopeptide; AP, alkaline phosphatase; SERMs, selective estrogen receptor modulators; FSH, follicle-stimulating hormone; PTH, parathyroid hormone.

Study and Year	Methods	Participants	Interventions	Outcomes	Key Findings
Berthold et al., 2004. Osteoporosis International [22] .	Randomized, double-blind, placebo-controlled, multicenter trial, 8 weeks.	49 postmenopausal women; Mean age: 61 ± 5 years; Mean time since menopause: 12.6 ± 8.8 years; Inclusion criteria: Age > 55 years, minimum 2 years post menopause, calcium intake > 1000 mg per day; Exclusion criteria: Metabolic bone disease (other than postmenopausal bone loss), malignancies, contraindications to statins, recent use of statins or other drugs affecting bone metabolism, fractures within the last 12 months, diabetes mellitus, complete lack of exercise, current smokers.	Atorvastatin 20 mg daily for 8 weeks; Comparator: Matching placebo for 8 weeks.	Bone formation markers: Intact osteocalcin, bone-specific alkaline phosphatase (BSAP), measured at baseline and 8 weeks; Bone resorption markers: C-telopeptide (CTx), intact parathyroid hormone, measured at baseline and 8 weeks; Undercarboxylated osteocalcin, measured at baseline and 8 weeks.	Atorvastatin had no statistically significant effect on bone formation or resorption markers in the overall group. However, in exploratory analyses, atorvastatin increased CTx and the CTx to osteocalcin ratio in women younger than 63 years and decreased these values in women older than 63 years.
Bjarnason et al., 2001. Osteoporosis International [23].	Randomized, open-label, single-center trial, 14 weeks. Computer-generated randomization was used with a 2:1 allocation ratio favoring fluvastatin.	68 women aged 65 years or older; Inclusion criteria: Hip and/or spine bone mineral density (BMD) below –2.0 SD of normal premenopausal women and a serum cholesterol concentration above 5.2 mmol/l; Exclusion criteria: BMI above 40 kg/m2, severe or chronic diseases, or medication known to interfere with the results of the study.	Fluvastatin 40 mg daily plus 500 mg Vitamin C daily for 12 weeks; Comparator: 500 mg Vitamin C daily for 12 weeks.	Bone formation markers: Serum total alkaline phosphatase (AP), serum osteocalcin (OC), measured at baseline, 4 weeks, and 12 weeks; Bone resorption markers: Urinary and serum CrossLaps (U-CTX and S-CTX), measured at baseline, 4 weeks, and 12 weeks; Serum lipids and lipoproteins, measured at baseline, 4 weeks, and 12 weeks.	Fluvastatin, given at a dose that resulted in the expected lipid-lowering effect, had no effect on bone formation markers. There was a slight, statistically significant, decrease in bone resorption markers in the fluvastatin group compared to baseline but not compared to the control group.
Bone et al., 2007. The Journal of Clinical Endocrinology & Metabolism [24].	Randomized, double-blind, placebo-controlled, multicenter, dose-ranging trial, 52 weeks.	626 postmenopausal women; Age range: 40–75 years; Inclusion criteria: LDL-C levels 130 - <190 mg/dL, Lumbar spine (L1-L4) BMD T-score between 0 and -2.5, postmenopausal with serum estradiol < 110 pmol/L and FSH > 30 IU/L; Exclusion criteria: Not specified in the excerpt.	Atorvastatin 10, 20, 40, or 80 mg daily for 52 weeks; Comparator: Matching placebo for 52 weeks; All participants also received calcium and vitamin D supplementation.	Primary outcome: Percent change in lumbar spine (L1-L4) BMD measured by DXA at baseline and 52 weeks; Secondary outcomes: Percent change in femoral neck, trochanter, total proximal femur BMD measured by DXA at baseline and week 52, lumbar spine (L1-L2) volumetric BMD measured by QCT in a subset of patients, bone turnover markers: serum N-telopeptide (NTx) and C-telopeptide (CTx) of type 1 collagen, osteocalcin, BSAP, N-terminal propeptide (P1NP) of type I procollagen, and urinary deoxypyridinoline (DPD), measured at baseline, 26 weeks, and 52 weeks.	Atorvastatin did not produce a significant change in BMD at any site or dose compared to placebo. No significant differences in bone turnover markers were observed between the atorvastatin and placebo groups.
Hsia et al., 2002. BMC Musculoskeletal Disorders [25].	Randomized, double-blind, placebo-controlled, dose-ranging trial, 12 weeks.	24 women; Inclusion criteria: Osteopenia assessed by broadband ultrasound attenuation; Exclusion criteria: Current use of estrogen, SERMs, calcitonin, bisphosphonates, or statins, positive pregnancy test, abnormal serum asparate aminotransferase levels.	Simvastatin 20 or 40 mg daily for 12 weeks; Comparator: Matching placebo for 12 weeks.	Bone formation marker: Bone-specific alkaline phosphatase (bone ALP), measured at baseline, 6 weeks, and 12 weeks; Bone resorption markers: Cross-linked N-telopeptides (NTx-I) and C-terminal propeptide (CTx-I) of type I collagen, measured at baseline, 6 weeks, and 12 weeks; Lipid profiles, measured at baseline and 12 weeks.	Simvastatin at either dose did not significantly affect bone formation or resorption markers compared to placebo.
Rejnmark et al., 2004. Journal of Bone and Mineral Research [26].	Randomized, double-blinded trial, one year, with a six-month follow-up. Randomization procedures not reported.	82 healthy postmenopausal women; Median age: 64 years; Median time since menopause: 19 years; Inclusion criteria: Osteopenia; Exclusion criteria: Not specified in the excerpt.	Simvastatin 40 mg daily for one year; Comparator: Matching placebo for one year; All participants received 400 mg/day elemental calcium supplements throughout the study period (1.5 years).	Primary outcome: Percent change in BMD at the lumbar spine and total hip at 52 weeks; Secondary outcomes: Changes in BMD at the femoral neck, forearm, and whole body at 52 weeks and 78 weeks; Biochemical indices of calcium homeostasis and bone metabolism, including plasma levels of cholesterol, PTH, and bone markers (bone-ALP, osteocalcin, and CTx), measured at baseline, 52 weeks, and 78 weeks.	Simvastatin had no effect on biochemical bone markers or BMD at the hip or spine. A statistically significant increase in BMD was observed in the simvastatin group at the forearm, but the authors do not think that this finding supports a general beneficial effect of simvastatin on bone.
Tanriverdi et al., 2005. European Journal of Obstetrics & Gynecology and Reproductive Biology [27].	Randomized, double-blind, parallel-group trial; Duration not reported.	60 postmenopausal women; Age: At least 50 years; Inclusion criteria: Postmenopausal, osteopenic or osteoporotic; Exclusion criteria: Previous or current treatment with hormone replacement therapy, bisphosphonates, selective estrogen receptor modulators, or calcium; Diseases or medications known to influence bone metabolism; Smoking; Alcohol consumption exceeding 20 g/day.	Atorvastatin 10 mg daily plus risedronate 5 mg daily; Comparator: Risedronate 5 mg daily.	BMD was measured at baseline and at the end of the study.	The combination of atorvastatin and risedronate resulted in a statistically significantly greater increase in vertebral BMD compared to risedronate alone.

Risk of bias in included studies

The risk of bias for the included studies was rigorously assessed using the Cochrane Risk of Bias tool. Three independent reviewers evaluated key domains independently. Discrepancies in individual assessments were resolved through consensus meetings, ensuring a robust and transparent evaluation process.

**Figure 2:**
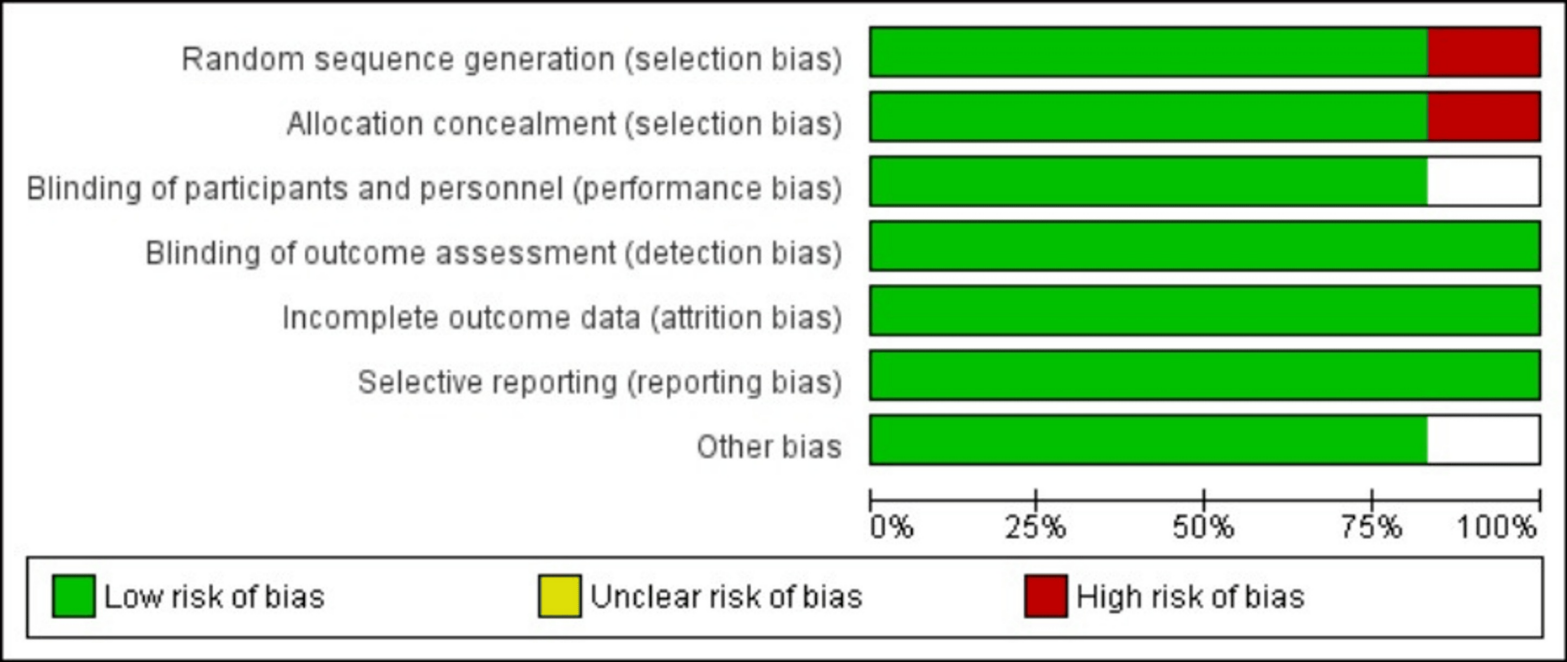
Risk of Bias of Included Studies

The risk of bias assessment indicates that most studies demonstrated a low risk of bias across key domains, ensuring robust methodological quality Figure 2. However, Tanriverdi et al. (2005) [27] showed a high risk in the domain of random sequence generation, and unclear risk was noted in allocation concealment and blinding domains for some studies, such as Berthold et al. (2004) [22] and Hsia et al. (2002) [25], which may impact the reliability of their findings [26].

Decreased incidence of overall fractures

None of the included studies specifically evaluated overall fracture reduction. While some studies indicated potential benefits of statins on bone metabolism and improvements in bone mineral density (BMD), the evidence lacked consistency and statistical significance in reducing fracture incidence. Consequently, pooling results for this outcome was not feasible.

However, Pena et al. (2015) analyzed data from the JUPITER trial, a large randomized, double-blind, placebo-controlled study of 17,802 participants with a 1.9-year median follow-up, assessed rosuvastatin's efficacy in reducing cardiovascular events and secondary outcomes like fracture risk [28]. Among postmenopausal women, rosuvastatin 20 mg daily showed no significant reduction in fracture risk (HR: 1.06; 95% CI: 0.88-1.28; p = 0.53). 

Percentage change in bone mineral density of the lumbar spine

**Figure 3:**
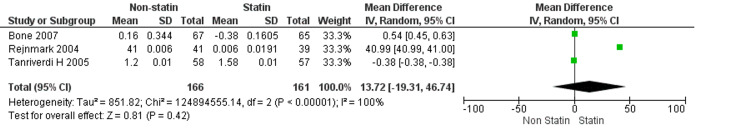
Percentage Change in Bone Mineral Density Lumbar Spine Data from Bone et al. (2007), Rejnmark et al. (2004), and Tanriverdi et al. (2005) were pooled, and the percentage change in bone mineral density of the lumbar spine was analyzed [24,26,27].

The pooled analysis demonstrates the percentage change in bone mineral density (BMD) of the lumbar spine among postmenopausal women receiving statin therapy compared to non-statin controls (Figure 3). The pooled mean difference was 13.72% (95% CI: -19.31 to 46.74), with no statistically significant overall effect observed (Z = 0.81, p = 0.42). Significant heterogeneity was detected across studies (I² = 100%, Tau² = 851.82, Chi² = 124894555.14, p < 0.00001). Individual studies reported variable outcomes [22,24,25], with Bone et al. (2007) [24] showing a modest positive effect (mean difference 0.54), Rejnmark et al. (2004) [26] reporting a larger positive effect (40.99), and Tanriverdi et al. (2005) [27] indicating a negative effect (-0.38).

Percentage change in bone mineral density at the femoral neck 

**Figure 4:**
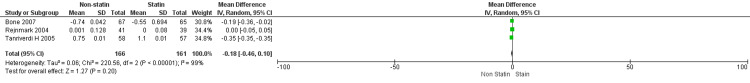
Percentage Change in Bone Mineral Density at the Femoral Neck Data from Bone et al. (2007), Rejnmark et al. (2004), and Tanriverdi et al. (2005) were pooled, and the percentage change in bone mineral density at the femoral neck was analyzed [24,26,27].

The pooled analysis evaluates the percentage change in bone mineral density (BMD) at the femur in postmenopausal women treated with statins compared to non-statin controls (Figure 4). The pooled mean difference was -0.18% (95% CI: -0.46 to 0.10), with no statistically significant effect observed (Z = 1.27, p = 0.20). Substantial heterogeneity was noted among the included studies (I² = 99%, Tau² = 0.06, Chi² = 220.56, p < 0.00001). Bone et al. (2007) reported a modest negative effect (-0.19), while Rejnmark et al. (2004) showed no meaningful difference (0.00), and Tanriverdi et al. (2005) demonstrated a slight negative effect (-0.35). The overall lack of significance and variability in study results suggests that statins may not have a consistent impact on femoral BMD, warranting further research to clarify these findings [24,26,27].

Forearm bone mineral density (BMD) findings

Among the included studies, only Rejnmark et al. (2004) reported significant improvements in forearm BMD with simvastatin treatment in postmenopausal women with osteopenia [26]. Forearm BMD increased by 1.1% at 52 weeks (p = 0.01) and 1.8% at 78 weeks (p = 0.02), compared to decreases in the placebo group. It is important to consider that this is an isolated finding, and more research is needed to confirm and understand this site-specific effect.

Secondary outcome: bone turnover markers

Changes In Bone-specific Alkaline Phosphatase (BSALP)

**Figure 5:**
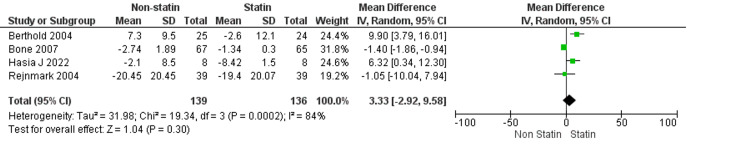
Changes in Bone-specific Alkaline Phosphatase (BSALP) Data from Berthold et al. (2004), Bone et al. (2007), Hsia et al. (2022), and Rejnmark et al. (2004) were pooled, and changes in bone-specific alkaline phosphatase (BSALP) were analyzed [22,24,25,26].

The pooled analysis assesses changes in anabolic bone markers, specifically bone-specific alkaline phosphatase (BSALP), in postmenopausal women treated with statins compared to non-statin controls (Figure 5). The pooled mean difference was 3.33 (95% CI: -2.92 to 9.58), with no statistically significant overall effect (Z = 1.04, p = 0.30). Moderate heterogeneity was observed across the studies (I² = 84%, Tau² = 31.98, Chi² = 19.34, p = 0.0002). Berthold et al. (2004) [22] showed a marked positive effect (mean difference 9.90), while Bone et al. (2007) [24] and Rejnmark et al. (2004) [26] indicated modest reductions (-1.40 and -1.05, respectively), and Hsia et al. (2022) [25] demonstrated a moderate positive effect (6.32). The variability among study findings highlights inconsistent effects of statins on BSALP levels, emphasizing the need for further research to establish their potential anabolic role in bone metabolism.

Changes From Baseline Osteocalcin

**Figure 6:**
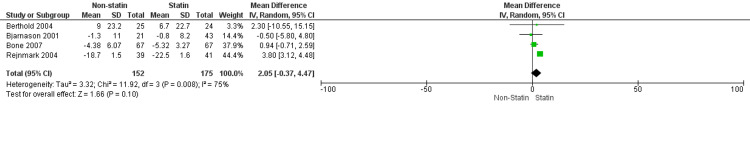
Changes From Baseline Osteocalcin Data from Berthold et al. (2004), Bjarnason et al. (2001), Bone et al. (2007), and Rejnmark et al. (2004) were pooled, and changes from baseline osteocalcin were analyzed [22-26].

The pooled analysis examines changes from baseline osteocalcin, an anabolic bone marker, in postmenopausal women treated with statins compared to non-statin controls (Figure 6). The pooled mean difference was 2.05 (95% CI: -0.37 to 4.47), suggesting no statistically significant effect (Z = 1.66, p = 0.10). Moderate heterogeneity was observed across studies (I² = 75%, Tau² = 3.32, Chi² = 11.92, p = 0.008). Berthold et al. (2004) showed a modest increase (mean difference 2.30), while Bjarnason et al. (2001) and Bone et al. (2007) reported minimal to no changes (-0.50 and 0.94, respectively), and Rejnmark et al. (2004) demonstrated a moderate increase (3.80). These findings indicate variability in the effect of statins on osteocalcin levels, warranting further exploration to elucidate their potential anabolic impact on bone turnover [22-26].

Changes in Resorptive Markers: C-Telopeptide of Type I Collagen (Ctx)

**Figure 7:**
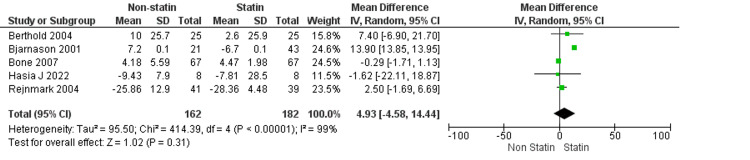
Changes in Resorptive Markers: C-Telopeptide of Type I Collagen (Ctx) Data from Berthold et al. (2004), Bjarnason et al. (2001), Bone et al. (2007), Hsia et al. (2022), and  Rejnmark et al. (2004) were pooled, and changes in resorptive marker C-telopeptide of type I collagen (Ctx) were analyzed [22-26].

The pooled analysis assesses changes in the resorptive bone marker C-telopeptide of type I collagen (CTX) in postmenopausal women treated with statins compared to non-statin controls (Figure 7). The pooled mean difference was 4.93 (95% CI: -4.58 to 14.44), indicating no statistically significant overall effect (Z = 1.02, p = 0.31). Significant heterogeneity was detected across studies (I² = 99%, Tau² = 95.50, Chi² = 414.39, p < 0.00001). Among the individual studies, Berthold et al. (2004) and Bjarnason et al. (2001) reported positive effects (mean differences 7.40 and 13.90, respectively), whereas Bone et al. (2007) and Hsia et al. (2022) demonstrated negligible to negative changes (-0.29 and -1.62, respectively), and Rejnmark et al. (2004) showed a modest increase (2.50). These findings reveal inconsistent effects of statins on CTX levels, suggesting the need for further research to clarify their role in modulating bone resorption [22-26].

Discussion

This systematic review and meta-analysis evaluated the efficacy of statins in treating osteoporosis in postmenopausal women, synthesizing data from six randomized controlled trials (RCTs) to assess their impact on bone mineral density (BMD), bone turnover markers, and fracture risk. The most notable finding was a statistically significant improvement in forearm BMD reported by Rejnmark et al. (2004), which showed increases of 1.1% at 52 weeks (p = 0.01) and 1.8% at 78 weeks (p = 0.02) with simvastatin compared to placebo. However, this effect was not observed at other skeletal sites, suggesting that the benefit may be site-specific [26].

For other skeletal regions, the results were mixed. Pooled analysis showed no statistically significant improvement in lumbar spine BMD (pooled mean difference: 13.72%; 95% CI: -19.31 to 46.74; Z = 0.81; p = 0.42) or femoral neck BMD (pooled mean difference: -0.18%; 95% CI: -0.46 to 0.10; Z = 1.27; p = 0.20). Individual studies such as Bone et al. (2007) reported minor positive effects of atorvastatin on lumbar spine BMD, whereas Hsia et al. (2002) and Tanriverdi et al. (2005) found no significant impact of statins on BMD or bone turnover markers [24,25,27]. These inconsistencies may be attributed to differences in study populations, statin types, treatment durations, and dosages, highlighting the challenges of drawing definitive conclusions from heterogeneous data.

Fracture risk reduction, a critical outcome in osteoporosis management, was not consistently demonstrated in the included RCTs. This contrasts with observational studies that have reported reductions in fracture risk associated with statin use. For example, while observational data suggest antiresorptive and anabolic effects of statins, this meta-analysis found no conclusive evidence of fracture risk reduction across the included trials.

Statins may serve as adjunctive therapies for patients with concurrent cardiovascular disease and osteoporosis due to their dual effects on lipid metabolism and bone remodeling [28,29]. While primarily indicated for cardiovascular risk reduction, site-specific BMD improvements (e.g., forearm BMD in Rejnmark et al. (2004)) and anti-inflammatory properties suggest potential bone health benefits [26]. However, inconsistent evidence for fracture risk reduction limits their use as standalone osteoporosis treatments. In patients already on statins for cardiovascular conditions, these secondary benefits may justify their continued use, especially in those at moderate fracture risk.

The analysis of bone turnover markers yielded variable results; while Berthold et al. (2004) reported subgroup-specific increases in bone-specific alkaline phosphatase (BSALP) and C-Telopeptide (CTX) levels, Bjarnason et al. (2001) and Rejnmark et al. (2004) did not replicate these findings [22,23,26].

Risk of bias assessments revealed methodological limitations in some studies, such as unclear allocation concealment and blinding, which may have influenced the variability in reported outcomes. The heterogeneity in study populations, statin types, doses, and treatment durations likely contributed to the variability in results.

The findings of this systematic review align with certain conclusions from earlier meta-analyses regarding the potential role of statins in osteoporosis treatment. For instance, previous reviews by An et al. (2017) and Uzzan et al. (2007) identified potential positive effects of statins on BMD, although these reviews did not specifically focus on postmenopausal women [5,10]. This consistency reinforces the notion that statins might exert anabolic or antiresorptive effects on bone, even though the clinical translation of these effects remains uncertain. The significant improvement in forearm BMD observed in Rejnmark et al. (2004) is consistent with these earlier analyses, suggesting possible site-specific benefits of statin therapy on bone metabolism [26].

However, this meta-analysis echoes the lack of consistent evidence for fracture risk reduction, as also reported in Bauer et al. (2004). Their analysis highlighted mixed results in RCTs regarding statins' impact on preventing fractures [15]. Similarly, the six RCTs included here failed to demonstrate statistically significant reductions in overall fracture incidence. These parallel findings suggest that while statins may influence BMD positively, this effect does not necessarily translate to a measurable clinical benefit in reducing fractures, a key outcome for osteoporosis management.

Despite areas of agreement, this meta-analysis highlights key differences from prior observational studies and meta-analyses, particularly regarding fracture risk reduction. Observational studies have frequently reported significant decreases in fracture risk associated with statin use, likely influenced by confounding factors such as healthier baseline characteristics of statin users or concurrent calcium and vitamin D supplementation. In contrast, none of the six included RCTs in this meta-analysis demonstrated consistent reductions in fracture incidence. Additionally, earlier meta-analyses, such as those by Hatzigeorgiou and Jackson (2005), suggested small but statistically significant improvements in lumbar spine and hip BMD with statin therapy, findings not corroborated by this review [16]. Instead, this analysis revealed mixed and statistically insignificant results for lumbar spine (pooled mean difference: 13.72%; 95% CI: -19.31 to 46.74) and femoral neck BMD (-0.18%; 95% CI: -0.46 to 0.10).

The observed discrepancies between the findings of these six studies and the broader literature may be attributed to several factors. First, differences in study design likely play a critical role. Many observational studies reporting fracture risk reductions did not account for confounders such as healthier baseline characteristics among statin users or co-administration of calcium and vitamin D, which could independently influence bone health. Conversely, the six included RCTs were more rigorously controlled, reducing the risk of bias but also narrowing the scope of outcomes assessed [22-27].

Second, variability in the types and doses of statins studied could contribute to differing results. Lipophilic statins, such as simvastatin and atorvastatin, were predominantly used in the included studies, whereas hydrophilic statins, such as pravastatin and rosuvastatin, which may have different effects on bone cells, were less represented. Furthermore, the relatively short treatment durations in some studies, such as Berthold et al. (2004) (8 weeks) and Hsia et al. (2002) (12 weeks), may have limited their ability to capture long-term effects on bone remodeling and fracture risk [22,25].

The populations studied also differed. The six trials included predominantly postmenopausal women with osteopenia or osteoporosis, whereas observational studies often included mixed populations, including men and individuals without diagnosed osteoporosis. This variation may have influenced the outcomes due to differences in baseline bone turnover rates and fracture susceptibility.

It is evident that the role of statins in osteoporosis remains controversial. While some earlier reviews, such as by Hatzigeorgiou and Jackson (2005) [16], suggested a modest protective effect of statins on BMD and fracture risk, more recent analyses, like An et al. (2017) [5], have highlighted significant heterogeneity and inconclusive results. This meta-analysis adds to the growing body of evidence that statins, while biologically plausible agents for bone health, do not consistently produce clinically meaningful outcomes in terms of fracture prevention or widespread BMD improvements in post-menopausal women.

Statins primarily act by inhibiting HMG-CoA reductase, a key enzyme in the mevalonate pathway responsible for cholesterol biosynthesis. This inhibition reduces the production of intermediates such as farnesyl pyrophosphate (FPP) and geranylgeranyl pyrophosphate (GGPP), which are essential for protein prenylation, a process critical for osteoclast function. By impairing osteoclast activity and inducing apoptosis, statins reduce bone resorption, thereby preserving bone density. Berthold et al. (2004) [22] supported this mechanism by demonstrating changes in bone resorption markers, such as CTX, though the effects varied with age and dosage [5-10,9,13,22].

Beyond reducing bone resorption, statins are believed to promote bone formation by stimulating mesenchymal stem cells (MSCs) and osteoblast activity. They enhance the expression of bone morphogenetic protein-2 (BMP-2), a key regulator of osteoblast differentiation, and increase endothelial nitric oxide synthase (eNOS), improving vascularization and bone remodeling [6]. Rejnmark et al. (2004) [26] reported significant site-specific increases in forearm bone mineral density (BMD) following simvastatin treatment, aligning with in vitro evidence of BMP-2 upregulation. However, these anabolic effects were not consistently observed across other skeletal sites or studies, underscoring variability in response and the need for further research to clarify these findings [5,6,8-10,13,26].

Statins exhibit promising molecular and cellular mechanisms that may influence BMD and fracture risk by inhibiting osteoclast activity and promoting osteoblast differentiation. These effects can help restore the imbalance in bone turnover characteristic of osteoporosis. Statins achieve this by targeting the mevalonate pathway, as described above. Additionally, their anti-inflammatory properties, achieved by modulating cytokines such as TNF-α and IL-6, may further contribute to decreased bone resorption [7]. However, despite these biologically plausible effects, clinical findings have been inconsistent [5,8-10,13]. For example, while Rejnmark et al. (2004) [26] reported significant improvements in forearm BMD, studies like Hsia et al. (2002) [25] and Bone et al. (2007) [24] found no significant effects on BMD or bone turnover markers in other skeletal regions.

Translating these molecular effects into clinically meaningful outcomes, such as fracture risk reduction, remains challenging. None of the six included studies consistently demonstrated reductions in fracture incidence, despite evidence from preclinical research showing dose-dependent increases in BMD and bone strength in animal models. The variability in clinical outcomes may stem from differences in statin type, dosage, treatment duration, and patient populations. For instance, Tanriverdi et al. (2005) [27] suggested potential benefits when atorvastatin was combined with risedronate, yet this combination's clinical impact remains limited compared to established therapies like bisphosphonates. Cost-effectiveness, prolonged treatment duration requirements, and the modest effects of statins on fracture prevention further complicate their integration into standard osteoporosis treatment protocols. Long-term, well-designed studies are essential to determine optimal regimens and clarify the potential of statins as adjunctive therapies in specific patient subgroups.

This systematic review and meta-analysis employed a comprehensive search strategy across multiple databases supplemented by manual searching, ensuring inclusion of relevant RCTs examining the efficacy of statins in osteoporosis management. The eligibility criteria were clearly defined and focused on postmenopausal women with osteoporosis and standardized interventions, enhancing the relevance and applicability of the findings. Data extraction and risk-of-bias assessments were independently conducted by multiple reviewers, reducing subjective bias and enhancing methodological rigor. The use of RevMan 5.0 for statistical analysis allowed for robust synthesis of data, including random-effects modeling to account for heterogeneity. Importantly, this review is novel in synthesizing findings from both bone turnover markers and BMD outcomes.

The outcomes of this meta-analysis provide a focused assessment of the therapeutic potential of statins specifically in postmenopausal women with osteoporosis, addressing a key gap in the existing literature. Previous meta-analyses, such as those by An et al. (2017) and Uzzan et al. (2007), have examined the broader use of statins in osteoporosis, including mixed populations of men and women [5,10]. By narrowing the scope to postmenopausal women, this analysis offers a more targeted evaluation of statins' efficacy in this high-risk group, where estrogen deficiency drives bone loss. This distinction allows for a clearer understanding of the role of statins in addressing osteoporosis-related outcomes in this specific population.

Despite its strengths, this review has notable limitations. The included studies exhibited significant heterogeneity in study populations, intervention types, and outcome measures, which impacts the generalizability of pooled results. For example, variations in the types of statins used (e.g., lipophilic versus hydrophilic) and treatment durations (ranging from 8 weeks to 1 year) likely contributed to the inconsistency in outcomes. Furthermore, none of the studies directly assessed long-term fracture risk, a critical metric for evaluating osteoporosis management efficacy.

Small sample sizes in several trials, such as those by Hsia et al. (2002) [25] and Berthold et al. (2004) [22], further reduced the statistical power needed to detect meaningful differences. Risk of bias assessments indicated methodological limitations in certain domains, including unclear allocation concealment and blinding in studies like Berthold et al. (2004) [22], which could have influenced the reliability of findings. Additionally, potential publication bias and the exclusion of non-English studies may have led to the omission of relevant research [30]. Finally, the relatively short follow-up durations in most trials were insufficient to evaluate the long-term effects of statins on bone remodeling and fracture prevention, emphasizing the need for extended, high-quality studies in the future.

To address these limitations, future research should prioritize large-scale, long-term RCTs to evaluate the effects of statins on fracture risk and bone health comprehensively. Studies should examine dose-response relationships, the differential effects of lipophilic and hydrophilic statins, and their interactions with other osteoporosis treatments. Furthermore, standardized reporting of bone turnover markers, BMD outcomes, and adverse events will enhance comparability across studies.

Future trials should employ rigorous designs with adequately powered sample sizes and extended follow-up durations. Control groups should include standard osteoporosis therapies, allowing for direct comparisons of statins as monotherapy or adjunctive treatments. Researchers should focus on clinically relevant outcomes, such as fracture incidence and quality of life, alongside BMD and bone turnover markers. Standardized dosing regimens and consistent outcome assessment methods, such as DXA and biochemical assays, are critical for robust data generation. Subgroup analyses based on age, baseline BMD, and comorbidities should be pre-specified to identify populations most likely to benefit from statin therapy.

Several critical questions remain unanswered, including the optimal type and dose of statins for bone health, their long-term safety in osteoporosis populations, and their cost-effectiveness compared to standard treatments. The potential synergistic effects of combining statins with other osteoporosis therapies, such as bisphosphonates or denosumab, require further investigation.

Although statins demonstrate promising biological mechanisms for enhancing bone health, this systematic review and meta-analysis found inconsistent evidence supporting their effectiveness in reducing fracture risk or significantly improving BMD in postmenopausal osteoporosis. As a result, their routine use in osteoporosis management cannot currently be recommended. Further robust, high-quality research is needed to provide definitive evidence and clarify their clinical utility in this context.

## Conclusions

This systematic review and meta-analysis aimed to evaluate the efficacy of statins in improving BMD and reducing fracture risk in postmenopausal women with osteoporosis, by synthesizing data from six RCTs, the pooled analysis revealed no statistically significant improvements in BMD at the lumbar spine (mean difference: 13.72%; 95% CI: -19.31 to 46.74; p = 0.42; I² = 100%) or femoral neck (mean difference: - 0.18%; 95% CI: -0.46 to 0.10; p = 0.20; I² = 99%). Significant heterogeneity was observed across studies, reflecting variability in intervention types and treatment durations. One study reported a 1.1% increase in forearm BMD with simvastatin at 52 weeks (p = 0.01); these findings were not generalizable to other skeletal sites. Moreover, none of the included studies demonstrated consistent reductions in fracture risk, underscoring the inconclusive clinical impact of statins in this context.

Statins may have adjunctive potential in specific populations, such as those with concurrent cardiovascular conditions, but variability in outcomes and lack of long-term fracture data limit their integration into current treatment guidelines. Furthermore, the moderate-to-low quality of evidence, as assessed using the GRADE framework, underscores the need for further high-quality RCTs with standardized protocols and extended follow-up durations. Future research should prioritize exploring dose-response relationships, identifying population-specific responses, and examining the synergy of statins with existing osteoporosis therapies. Additionally, studies should evaluate clinically relevant outcomes, such as fracture reduction, quality of life, and long-term safety, to provide robust evidence for integrating statins into osteoporosis treatment strategies.

## Appendices

**Table 3:** Data Extraction Tool

Data Extraction Field	Data Collected from each study
Information about data extraction from reports	
Name of data extractors	
Date of data extraction	
Identification features of report	
Eligibility criteria	
Confirm eligibility of the study for the review	
Reason for exclusion	
Study methods	
Study design	
Single or multicenter study	
If multicenter, number of recruiting centers	
Recruitment and sampling procedures used (including at the level of individual participants and clusters/sites if relevant)	
Enrolment start and end dates; length of participant follow-up	
Details of random sequence generation	
Allocation sequence concealment	
Masking for randomized trials	
Methods used to prevent and control for confounding( selection biases, and information biases )	
Methods used to prevent and address missing data	
Unit of analysis (e.g., individual participant, clinic, village, body part)	
Statistical methods used if computed effect estimates are extracted from reports, including any covariates included in the statistical model	
Likelihood of reporting and other biases	
Source(s) of funding or other material support for the study	
Authors' financial relationships and other potential conflicts of interest	
Participants	
Setting	
Region(s) and country/countries from which study participants were recruited	
Study eligibility criteria, including diagnostic criteria	
Characteristics of participants at the beginning (or baseline) of the study (e.g., age, sex, comorbidity, socio-economic status)	
Intervention	
Description of the intervention(s) and comparison intervention(s), ideally with sufficient detail for replication	
Components, routes of delivery, doses, timing, frequency, intervention protocols, length of intervention	
Factors relevant to implementation (e.g., staff qualifications, equipment requirements)	
Integrity of interventions (i.e., the degree to which specified procedures or components of the intervention were implemented as planned)	
Description of co-interventions	
Definition of 'control' groups (e.g., no intervention, placebo, minimally active comparator, or components of usual care)	
Components, dose, timing, frequency	
Outcomes	
For each pre-specified outcome domain	
Whether there is evidence that the outcome domain was assessed	
Measurement tool or instrument (including definition of clinical outcomes or endpoints); for a scale, name of the scale, upper and lower limits, and whether a high or low score is favorable, definitions of any thresholds if appropriate	
Specific metric	
Method of aggregation (e.g., mean and standard deviation in each group, or proportion of people)	
Timing of outcome measurements (e.g., assessments at end of eight-week intervention period, events occurring during the eight-week intervention period)	
Adverse outcomes need special attention depending on whether they are collected systematically or non-systematically (e.g., by voluntary report)	
Results	
For each group, and for each outcome at each time point: number of participants randomly assigned and included in the analysis; and number of participants who withdrew, were lost to follow-up or were excluded (with reasons for each)	
Summary data for each group (e.g., 2×2 table for dichotomous data; means and standard deviations for continuous data)	
Between-group estimates that quantify the effect of the intervention on the outcome, and their precision (e.g., risk ratio, odds ratio, mean difference)	
If subgroup analysis is planned, the same information would need to be extracted for each participant subgroup	
Miscellaneous	
Key conclusions of the study authors	
Reference to other relevant studies	
